# A comparison of the effects of Temporin atomizable mucosal liquid dressing vs. routine skin care on wound healing in critically ill patients: a retrospective analysis

**DOI:** 10.3389/fmed.2026.1794422

**Published:** 2026-05-18

**Authors:** Xueran Cui, Liqing Li, Qian Liu

**Affiliations:** 1 Critical Care Medicine Department, The Second Hospital of Baoding, Baoding City, Hebei, China; 2 Department of Neurosurgery, The Second Hospital of Baoding, Baoding City, Hebei, China

**Keywords:** anti-infective agents, critical illness, local, pressure injury, skin care, wound healing

## Abstract

**Objective:**

This study aimed to compare the effects of Temporin atomizable mucosal liquid dressing vs. routine skin care on healing outcomes (healing rate and healing time) in critically ill patients with existing Stage 2 pressure injuries or oral injuries.

**Methods:**

This retrospective analysis included 135 critically ill patients admitted between November 2024 and July 2025, allocated to a Temporin group (*n* = 67) or a control group (*n* = 68) based on the intervention received. The Temporin group received skin care three times daily using Temporin atomizable mucosal liquid dressing, whereas the control group received routine care (normal saline for oral injuries; povidone–iodine for external lesions). The intervention lasted 14 days. Primary outcomes were healing rate and healing time; the secondary outcome was adverse reactions. Between-group comparisons were performed using t-test, Mann–Whitney U test, or χ^2^ test, as appropriate. Multivariable regression analyses were adjusted for age, APACHE II score, and lesion location. Effect sizes are reported with 95% confidence intervals (CIs).

**Results:**

After 14 days of intervention, the skin injury healing rate in the Temporin group (97.0%) was significantly higher than that in the control group (69.1%) (adjusted risk ratio = 1.40, 95% CI: 1.18–1.66; *p* < 0.01). The mean healing time in the Temporin group was 5.2 days, significantly shorter than 7.4 days in the control group (adjusted mean difference = −2.1 days, 95% CI: −3.0 to −1.2; *p* < 0.0001). No adverse reactions were reported in either group.

**Conclusion:**

The use of Temporin atomizable mucosal liquid dressing was associated with higher healing rates and shorter healing times in critically ill patients. These findings suggest a promising alternative to traditional nursing methods, although further prospective validation is warranted.

## Introduction

Critically ill patients face a high risk of various skin complications due to the complexity and severity of their conditions (1, 2). Among these, pressure injuries and moisture-associated dermatitis are common issues that pose additional challenges to patient recovery (3, 4). Pressure injuries refer to localised damage to the skin and/or underlying tissue, usually over a bony prominence, caused by prolonged pressure or pressure combined with shear (5, 6). In critically ill patients, extended bed rest and limited mobility result in sustained external pressure on the skin, significantly increasing the risk of tissue damage (7). Furthermore, the skin barrier function in critically ill patients is often compromised, with a reduced ability to retain moisture, making them prone to problems such as skin dryness and desquamation, thereby increasing the risk of moisture-associated dermatitis (8–10).

Although existing routine skin care methods, such as disinfection with antiseptics and application of normal saline, provide some level of cleansing and moisturising, they still have limitations in critically ill patients (11–13). Traditional skin care approaches may not fully address the unique skin needs of this population. For instance, in bedridden patients, simple cleaning and moisturising may be insufficient to effectively prevent pressure injuries (14). Additionally, some conventional skin care products may be excessively irritating to the fragile skin of critically ill patients, potentially causing allergic reactions or other adverse skin conditions (15).

Temporin atomizable mucosal liquid dressing is a clinically used skin care product, especially suitable for sensitive and damaged skin. Its main ingredients are purified water, antiseptics (cetylpyridinium chloride), moisturisers (hyaluronic acid), and pH buffers (citrates or phosphates) to maintain a mildly acidic environment compatible with the skin’s physiology. It is used for cleaning, disinfecting, and moisturising the skin and mucous membranes. It is often used in intensive care units (ICUs) and other environments to prevent and manage conditions such as pressure injuries, incontinence-associated dermatitis, peristomal skin irritation, and oral mucositis. As a novel skin care product, it possesses unique properties that may offer new advantages for skin care in critically ill patients. Its pH-balanced design helps maintain the acid–base balance on the skin surface, thereby preventing skin problems caused by pH imbalance. Simultaneously, this rinse solution also exhibits certain antibacterial properties, effectively reducing bacterial colonisation on the skin surface and lowering the risk of infection.

This study aims to compare the effectiveness of Temporin atomizable mucosal liquid dressing vs. traditional care in promoting healing of existing skin injuries (pressure injuries and oral injuries) in critically ill patients, thereby providing evidence for an effective treatment option in clinical practice.

## Methods

### Study design

This study was a single-centre, retrospective cohort analysis. Data were collected from The Second Hospital of Baoding electronic medical record system for critically ill patients admitted between November 2024 and July 2025. The study was approved by the hospital ethics committee. This study adhered to the principles of the Declaration of Helsinki and was granted a waiver for informed consent (only de-identified data were used).

Patients were allocated to either the Temporin group or the control group based on the skin care protocol documented in their electronic medical records during their stay in the ICU. The allocation was determined by the treating clinical team as part of routine care, not by the study investigators. Both treatment options—Temporin Atomised Temporin atomizable mucosal liquid dressing and routine care (normal saline for oral injuries and povidone–iodine for external skin lesions)—were concurrently available throughout the entire study period. There was no time-based implementation (i.e., no before/after period), and no hospital protocol dictated which patients should receive one treatment rather than the other. The choice was made at the discretion of the attending physicians and nurses, reflecting real-world clinical practice.

**Figure 1:**
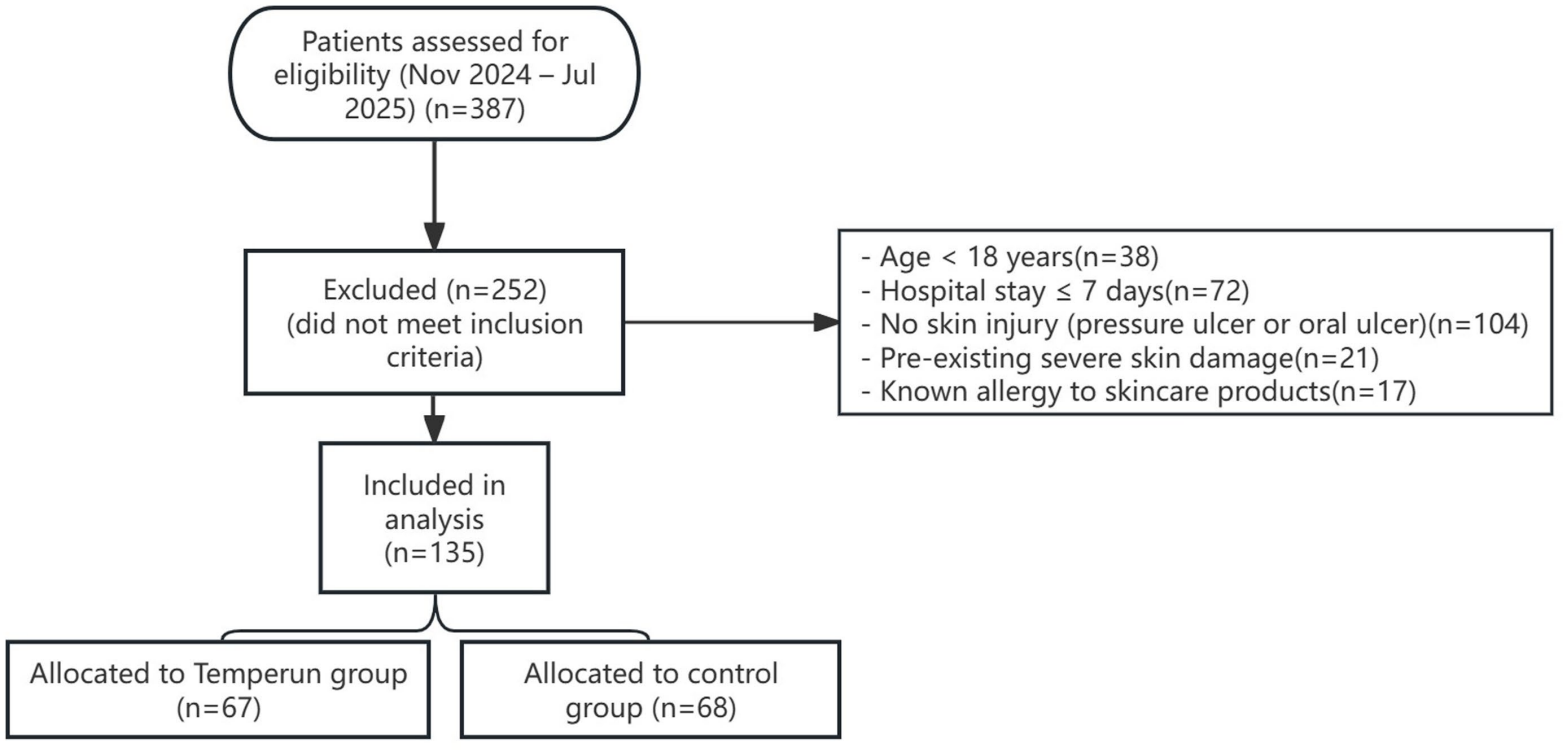
Flow diagram of patient selection and group allocation.

To minimise selection bias, strict inclusion and exclusion criteria were applied, ensuring that all eligible patients during the study period were considered for analysis. Data were extracted consecutively from the electronic medical record system without preselection based on outcomes. The baseline characteristics of the two groups were compared to assess comparability, and no statistically significant differences were observed in age, gender, length of stay, or Acute Physiology and Chronic Health Evaluation (APACHE) II scores, suggesting that selection bias due to observable confounders was limited. A Strengthening the Reporting of Observational Studies in Epidemiology (STROBE)-style flow diagram (Figure 1) illustrates the patient selection process.

### Study participants

#### Inclusion criteria

- Adult ICU patients (aged ≥18 years).
- Hospital stay >7 days (to ensure sufficient exposure to the intervention).
- Classified as Stage 2 (partial-thickness skin loss) according to the international National Pressure Ulcer Advisory Panel (NPUAP) staging system (16) or presence of oral mucosal pressure injuries (17). Only one pressure injury wound was selected from each patient as a representative for data collection.
- Receiving device fixation (catheters/breathing masks) or requiring high-frequency skin cleaning (≥2 times/day).

#### Exclusion criteria

- Pre-existing severe skin damage (e.g., deep injuries or extensive skin breakdown) before enrolment, to avoid interference from previous skin conditions on the study results.
- Known history of allergies, particularly to the ingredients of Temporin atomizable mucosal liquid dressing or the routine skin care products used in the study, to prevent allergic reactions from affecting skin condition assessment.

### Study termination criteria

The study termination criteria were as follows: (1) occurrence of any severe adverse reaction (e.g., systemic allergic reaction or severe localised irritation) clearly attributable to the study intervention, (2) a significant deterioration in the patient’s overall condition requiring discontinuation of standard skin care protocols, or (3) voluntary withdrawal of the patient or their legal representative from the study.

#### Sample size calculation

To assess whether the available sample size provided adequate statistical power to detect clinically meaningful differences between groups, a *post hoc* power analysis using G*Power 3.1.9.7 (Heinrich Heine University, Düsseldorf, Germany) was performed (18). The calculation was based on the primary outcome variable, the 14-day healing rate.

Assuming a control group healing rate of approximately 65% (based on historical data from routine care at the study institution) and a clinically meaningful absolute improvement of 30% (to 95%) in the Temporin group, with a two-sided significance level *α* = 0.05 and power (1 - *β*) = 0.80, the required sample size was calculated to be ≥67 patients per group. This effect size was considered clinically meaningful, as a 30% improvement in healing rate would represent a substantial benefit in ICU wound management and aligns with preliminary clinical observations, suggesting superior healing with Temporin solution.

With the final analytic sample of 67 patients in the Temporin group and 68 patients in the control group, the study achieved 80% power to detect the hypothesised 30% absolute difference in healing rates. However, it is important to note that this represents a *post hoc* justification of sample size adequacy rather than a prospective power calculation, consistent with the retrospective nature of the study. The sample size may be insufficient for detecting smaller effect sizes, conducting subgroup analyses, or identifying rare adverse events.

### Grouping and intervention

#### Temporin group

Patients used Temporin atomizable mucosal liquid dressing for skin cleaning and care. The frequency of use was set at three times daily based on the product instructions and clinical experience. The operational procedure included ensuring the skin was clean and dry before application, applying an appropriate amount of Temporin solution evenly to the skin surface, gently massaging until fully absorbed, and avoiding vigorous rubbing that could cause skin damage. The entire intervention period was 14 days to fully assess its impact on skin integrity.

#### Control group

Patients received routine skin care products according to the hospital’s standard operating procedures. Oral injuries were treated with normal saline cleansing three times daily, and external skin lesions were treated with povidone–iodine disinfection three times daily. The intervention period was also 14 days, consistent with the Temporin group, to facilitate comparison of effectiveness.

Patients were assigned to either the Temporin group or the control group based on the skin care protocol documented in their electronic medical records during their ICU stay. The assignment was determined by the treating clinical team as part of routine care, not by the study investigators. No specific randomisation or allocation sequence was applied, consistent with the retrospective nature of the study. The two groups were treated concurrently during the study period (November 2024 to July 2025), and there was no change in the skin care protocols for either group during this time. All interventions followed the hospital’s standard operating procedures, which remained consistent throughout the study period. Therefore, no temporal bias or protocol shift affecting group assignment or intervention delivery was identified.

### Outcome measures

#### Primary outcomes

- Healing

Healing was defined as complete re-epithelialisation of the skin lesion with the absence of drainage, erythema, and tenderness, as documented by the attending clinician at the end of the 14-day intervention period. All assessments were performed using standardised criteria based on the NPUAP staging system (16), and all assessors received training in wound evaluation before the study. Due to the retrospective design, assessors were not blinded to group assignment, which may introduce detection bias. Inter-rater reliability was not formally assessed because data were extracted from routine clinical records; future prospective studies should include blinded outcome assessors and evaluate inter-rater reliability to enhance validity.

- Wound Healing Time

Healing time was defined as the number of days from the start of the intervention to the first documented complete wound closure.

#### Secondary outcomes

- Any adverse reactions (e.g., allergies and irritation) during the intervention period were recorded. The time of occurrence, symptoms, severity, and management measures were documented in detail. Safety was evaluated by comparing the incidence of adverse reactions between the two groups.

### Assessment tools and procedures

- Skin assessment criteria: The international NPUAP staging system was used to assess skin injuries (16). This system classifies skin injuries into different stages based on depth, tissue type, and characteristics, providing a standardised tool for accurate assessment. Assessors conducted comprehensive skin evaluations before the intervention, during the intervention (weekly), and at the end of the intervention, recording the results.
- Data collection time points: Baseline data (before intervention) on patient demographics and skin condition were collected as the starting state, including age, sex, expected hospital stay, and APACHE II score. The APACHE II score is a widely used ICU prognostic system that quantifies disease severity by assessing 12 routine physiological variables (e.g., vital signs and laboratory values), age, and chronic health status. Scores range from 0 to 71, with higher scores indicating greater disease severity and higher risk of mortality.
- Location of skin lesions: A comprehensive assessment was repeated at the end of the intervention (day 14) to collect data related to primary and secondary outcomes for evaluating the effect over the entire intervention period. Additionally, interim assessments were conducted during the intervention (e.g., day 7) to identify and address any potential issues promptly, ensuring the smooth progress of the study.

### Statistical methods

Data analysis was performed using SPSS 26.0 (IBM Corp., Armonk, NY, USA) (19, 20). For continuous variables (e.g., age, APACHE score, and healing time), normality was tested first. If data were normally distributed, they were expressed as mean ± standard deviation, and comparisons between groups were made using independent samples *t*-tests. If data were not normally distributed, they were expressed as M(IQR), and comparisons between groups were made using the Mann–Whitney U test. For categorical variables (e.g., gender and skin injury incidence), frequency and percentage (*n*[%]) were used, and comparisons between groups were made using the *χ*^2^ test or Fisher’s exact test. To control for potential confounding factors, multivariate regression analysis was performed.

- For the binary outcome (healing rate at 14 days), binary logistic regression was used to calculate adjusted odds ratios (ORs) with 95% confidence intervals (CIs), and modified Poisson regression with robust error variance was used to obtain adjusted risk ratios (RRs) with 95% CIs. Adjustments included age, APACHE II score, and lesion location (lesion sites were collapsed into three categories: oral injury, facial lesion, and other sites due to the small sample size in some subgroups).
- For healing time, three analytical approaches were employed:

*Descriptive and non-parametric comparison*: Since the healing time was non-normally distributed (confirmed using the Shapiro–Wilk test), the Mann–Whitney U test was used for unadjusted between-group comparisons.

*Survival analysis*: To account for censoring (e.g., patients discharged or transferred before complete healing), a Kaplan–Meier survival analysis was conducted, and groups were compared using the log-rank test. Median healing time with 95% CIs was reported for each group. A Cox proportional hazards model was used to estimate the hazard ratio (HR) with 95% CIs, adjusting for age, APACHE II score, and lesion location.

*Linear regression for adjusted mean difference*: Although healing time was not normally distributed, the residuals from the linear model were approximately normally distributed; therefore, a multiple linear regression model was fitted to estimate the adjusted mean difference in healing time (with 95% CIs) between groups, adjusting for the same covariates.

- Effect sizes are reported alongside *p*-values to provide a clearer interpretation of clinical relevance.

Given the limited sample size, propensity score matching was not performed; however, sensitivity analysis was conducted to test the robustness of the results. A two-sided *p*-value <0.05 was considered statistically significant.

To control for potential confounding, baseline demographic and clinical characteristics (age, gender, APACHE II score, and lesion location) were compared between groups. Due to the retrospective design, multivariable regression analysis was not pre-specified; however, exploratory analyses suggested that between-group differences in disease severity (APACHE II) and lesion type distribution were not statistically significant. Future prospective studies should employ propensity score matching or multivariable adjustment to better control for confounding.

### Adverse event monitoring and definition

Adverse events were actively monitored throughout the 14-day intervention period using a standardised protocol. The following procedures were implemented:

- Daily skin assessment: Attending nurses performed daily visual inspections of all treated areas, documenting any signs of local irritation (erythema, rash, pruritus, swelling, and pain) or systemic reactions.
- Standardised definitions: Adverse events were classified according to the Common Terminology Criteria for Adverse Events version 5.0. Local skin reactions were graded as mild (Grade 1: asymptomatic or mild symptoms), moderate (Grade 2: localised intervention indicated), or severe (Grades 3–5: requiring discontinuation or hospitalisation).
- Reporting protocol: Any suspected adverse reaction was recorded in the electronic medical record, including time of onset, severity, duration, and management measures. All events were reviewed by the attending physician and documented in the case report form.
- Causality assessment: The relationship between the intervention and any adverse event was assessed by the clinical team as “unrelated,” “possibly related,” “probably related,” or “definitely related” based on temporal association and alternative explanations.

Given the retrospective design, adverse event data were extracted from routine clinical documentation; therefore, underreporting or incomplete documentation cannot be excluded.

## Results

### Baseline characteristics of study participants

A total of 387 critically ill patients admitted during the study period were initially assessed. After applying inclusion and exclusion criteria, 135 patients were included in the analysis: 67 in the Temporin group and 68 in the control group. The flow of participants through the study is shown in Figure 1. The two groups were comparable in terms of baseline characteristics, such as age, gender, expected length of hospital stay, and APACHE score (Table 1). Specifically, the mean age was 70.09 years in the Temporin group and 68.34 years in the control group, with no statistically significant difference (*p* = 0.8432). Regarding gender distribution, the Temporin group had 46 men (68.7%) and 21 women (31.3%); the control group had 42 men (61.8%) and 26 women (38.2%), with no statistically significant difference in gender distribution (*p* = 0.7511). The expected length of hospital stay also showed no significant difference between groups (Temporin group: 13.36 days, control group: 12.32 days). There was some difference in APACHE scores between groups (Temporin group: 33.26 ± 5.22, control group: 38.38 ± 7.78), but this difference did not reach statistical significance (*p* = 0.1254).

**Table 1:** Baseline characteristics and outcomes of patients in TempRun and control groups.

Variable	TempRun group (*n* = 67)	Control group (*n* = 68)	*p*-value
Age (year)	70.09 ± 14.54	68.34 ± 18.33	0.8432
Sex			0.7511
Male	46 (68.7%)	42 (61.8%)	
Female	21 (31.3%)	26 (38.2)	
Expected hospital stay (days)	13.36 ± 7.55	12.32 ± 7.78	0.0894
APACHE score	33.26 ± 5.22	38.38 ± 7.78	0.1254
Location of skin lesions			0.0915
Oral injuries	22 (32.8%)	17 (25.0%)	
Facial lesions	18 (26.9%)	31 (45.6%)	
Tracheostomy lesions	2 (3.0%)	2 (2.9%)	
Genital and perineal lesions	10 (14.9%)	4 (5.9%)	
Limb lesions	6 (9.0%)	4 (5.9%)	
Head and neck lesions	3 (4.4%)	2 (2.9%)	
Waist and abdominal lesions	6 (9.0%)	8 (11.8%)	

### Results of multivariate regression analysis

To further adjust for the influence of baseline disease severity and lesion location on the outcomes, multivariate regression analysis was performed. After adjustment for age, APACHE II score, and lesion location, the 14-day healing rate remained significantly higher in the Temporin group than in the control group (adjusted OR = 8.2, 95% CI 2.9–23.1, *p* < 0.001; adjusted RR = 1.40, 95% CI 1.18–1.66, *p* < 0.001), and the mean healing time was significantly shorter (adjusted mean difference = −2.1 days, 95% CI –3.0 to −1.2, *p* < 0.001). These findings indicate that the beneficial effect of Temporin solution in promoting skin lesion healing is independent of the measured confounders mentioned above. However, due to the limitations of the retrospective design and potential unmeasured confounders, the results should be interpreted with caution.

### Skin injury incidence

There were some differences in the distribution of skin lesion locations between the two groups. The incidence of oral injuries was 32.8% in the Temporin group vs. 25.0% in the control group; facial skin lesions were 26.9 and 45.6%, respectively; genital and perineal skin lesions were 14.9 and 5.9%, respectively; and limb skin lesions were 9.0 and 5.9%, respectively. Although the incidence rates for some lesion locations differed between groups, the overall difference in skin injury incidence did not reach statistical significance (*p* = 0.0915) (see Table 1). Given the physiological differences between the oral mucosa and epidermis, a subgroup analysis was performed. In the Temporin group, oral injuries accounted for 32.8% of lesions, whereas in the control group, they accounted for 25.0%. Due to the relatively small sample size within each anatomical subgroup, statistical comparisons of healing rates stratified by lesion location were not feasible. However, it should be noted that the moist environment of the oral cavity and the drier conditions of the external skin may influence healing dynamics differently; therefore, the aggregated healing data presented below should be interpreted with this anatomical heterogeneity in mind.

### Wound healing time

After the 14-day intervention, the healing rate in the Temporin group was 97.0%, significantly higher than the 69.1% in the control group (adjusted OR = 8.2, 95% CI: 2.9–23.1, *p* < 0.001; adjusted RR = 1.40, 95% CI: 1.18–1.66, *p* < 0.001). Furthermore, the average healing time was 5.2 days in the Temporin group compared with 7.4 days in the control group (adjusted mean difference = −2.1 days, 95% CI: −3.0 to −1.2, *p* < 0.001). As the healing time was not normally distributed, the M(IQR) healing time was 5.1 (3.7–6.2) days in the Temporin group and 7.2 (5.1–8.9) days in the control group (Mann–Whitney U test, *p* < 0.001). The difference in healing time between the two groups was statistically significant (*p* < 0.0001), indicating a significant advantage for the Temporin group in accelerating skin injury healing (see Table 2).

**Table 2:** The statistics of the patients’ healing rate, healing time, and incidence of adverse reactions after intervention.

Variable	TempRun group (*n* = 67)	Control group (*n* = 68)	Effect size (95% CI)	*p*-value
Healing rate after 14 days of intervention	65 (97.0%)	47 (69.1%)	Adjusted RR = 1.40 (1.18–1.66)	*p* < 0.01
Healing time of injuries (days)	5.1 (3.7–6.2)	7.2 (5.1–8.9)	Adjusted mean difference = −2.1 (−3.0 to −1.2)	*p* < 0.0001
Incidence of adverse reactions	0	0	–	/

To explore whether the therapeutic effect of Temporin solution varied between oral and external lesions, exploratory subgroup analyses were conducted. Among patients with oral injuries, the mean healing time in the Temporin group was 4.8 [3.7–5.9] days (*n* = 22) vs. 6.9 [5.2–8.9] days in the control group (*n* = 17). Among patients with external skin lesions (e.g., pressure injuries and facial lesions), the mean healing time was 5.4 [4.1–6.2] days in the Temporin group (*n* = 45) vs. 7.6 [5.7–8.9] days in the control group (*n* = 51). Although both subgroups favoured the Temporin intervention, the magnitude of benefit appeared slightly more pronounced in oral lesions. However, due to the small sample sizes and the retrospective design, these differences should be interpreted with caution and warrant further prospective validation.

Kaplan–Meier analysis revealed a significantly faster healing trajectory in the Temporin group (log-rank test, *p* < 0.001). The median time to healing (with 95% CI) was 5.0 days (95% CI: 4.5–5.5) in the Temporin group and 7.0 days (95% CI: 6.2–7.8) in the control group. In the Cox proportional hazards model adjusted for age, APACHE II score, and lesion location, the HR for healing in the Temporin group compared with the controls was 2.3 (95% CI: 1.6–3.3, *p* < 0.001), indicating that patients in the Temporin group had more than twice the likelihood of healing at any given time point.

### Incidence of adverse reactions

During the 14-day intervention period, no adverse reactions were documented in either the Temporin group or the control group based on the available clinical records. Specifically, no cases of local irritation, allergic reaction, or discontinuation due to skin intolerance were reported in either group.

## Discussion

This retrospective analysis compared the effects of Temporin atomizable mucosal liquid dressing and routine skin care on wound healing in critically ill patients. The results showed that the Temporin group had a significantly higher healing rate after the 14-day intervention (97.0% vs. 69.1%, adjusted RR = 1.40, 95% CI: 1.18–1.66, *p* < 0.001) and a significantly shorter healing time (5.2 days vs. 7.4 days, adjusted mean difference = −2.1 days, 95% CI: −3.0 to −1.2, *p* < 0.0001), suggesting that the use of Temporin atomizable mucosal liquid dressing may be associated with improved healing outcomes in this patient population. However, given the retrospective nature of this study and the baseline differences in disease severity (as reflected by APACHE II scores), these findings should be interpreted as hypothesis-generating rather than confirmatory. No adverse reactions were documented in either group during the 14-day intervention period. Although this observation is reassuring, it should be interpreted with caution, given the limited sample size (*n* = 135) and short follow-up duration, which are insufficient to detect rare or delayed adverse events. The absence of documented reactions may also reflect under-reporting or incomplete documentation inherent to retrospective designs.

The pH-balanced design of Temporin atomizable mucosal liquid dressing helps maintain the acid–base balance of the skin surface. The normal skin pH is typically 4.5–6.0; this slightly acidic environment can inhibit bacterial growth and reproduction while helping to maintain skin barrier integrity (21–23). In critically ill patients, skin barrier function is often weakened and easily disturbed by external factors (24, 25). Using Temporin atomizable mucosal liquid dressing can effectively avoid skin problems caused by pH imbalance, thereby reducing the risk of skin infection (26). The rinse solution also possesses certain antibacterial properties, effectively reducing bacterial colonisation on the skin surface. In critically ill patients with compromised immune function, the skin can easily become an entry point for bacterial infections. Using care products with antibacterial properties can reduce bacterial colonisation on the skin surface, lowering the incidence of infection. In this study, the significantly shorter healing time in the Temporin group may be related to the antibacterial effect of the rinse solution, reducing interference from infection on wound healing.

An important consideration in interpreting these results is the inherent physiological difference between oral mucosal healing and epidermal healing. The oral mucosa is characterised by a moist environment, rapid epithelial turnover, and a rich vascular supply, which typically facilitates faster healing than keratinised epidermis. By contrast, epidermal wounds, such as Stage 2 pressure injuries, heal in a relatively dry environment and are more susceptible to desiccation and external friction. The Temporin atomizable mucosal liquid dressing, containing hyaluronic acid and cetylpyridinium chloride, may exert dual effects: maintaining a moist environment conducive to mucosal healing while also providing a protective barrier for external skin. However, the aggregated data in this study do not allow for a direct comparison of healing mechanisms across tissue types. Future studies should stratify outcomes by lesion location and consider using histologic or biomarkers to assess tissue-specific responses. Readers should therefore avoid conflating the product’s effect on oral injuries with its effect on pressure injuries, as these represent distinct wound healing environments.

In addition, although the two groups were generally comparable at baseline, there was a difference in the APACHE II scores; the Temporin group had a mean score of 33.26, whereas the control group had a mean score of 38.38. Although this difference was not statistically significant, it suggests that the Temporin group may have been somewhat less severely ill at baseline. This could have contributed to the better healing outcomes observed in this group. In other words, the higher healing rate in the Temporin group may be partly explained by their better overall condition rather than the intervention alone. Future studies with prospective designs and larger sample sizes are needed to confirm these findings and better control for such confounding factors. It should be noted that the control group received povidone–iodine for disinfection of external skin lesions, a practice that, although standard in some clinical settings, has been associated with cytotoxic effects on healing tissues. This may have contributed to the relatively lower healing rate and longer healing time observed in the control group. Therefore, the superior outcomes in the Temporin group should be interpreted with caution, as they may partly reflect the limitations of the control intervention rather than an inherent advantage of the Temporin solution alone. Future studies should consider using a more neutral or evidence-based comparator to better isolate the therapeutic effect of Temporin atomizable mucosal liquid dressing.

This study has several methodological strengths. First, it focused on a clinically relevant ICU population at high risk for skin integrity compromise. Second, primary outcomes were clearly defined using objective criteria, and all wounds were assessed using the internationally recognised NPUAP staging system. Third, the observed effect sizes were large and statistically robust, with an adjusted RR of 1.40 for healing and a mean reduction in healing time of 2.1 days. Fourth, the intervention frequency was standardised across both groups (three times daily), reducing performance variability. Finally, the study adhered to ethical standards with transparent reporting and institutional approval.

Several limitations inherent to the retrospective design should be considered when interpreting our findings. First, selection bias may have occurred because patients were not randomly assigned to the intervention groups; allocation was determined by clinicians’ real-world practice based on product availability or physician preference. Although the two groups were treated concurrently during the entire study period (November 2024–July 2025), which minimises temporal bias, the lack of blinding introduces performance and detection bias. Patients, caregivers, and outcome assessors were aware of the skin care intervention received, which may have influenced behaviours and expectations. For instance, nursing staff may have applied Temporin more meticulously or provided additional skin care to patients in the Temporin group, thereby enhancing outcomes independent of the product’s intrinsic effects. Conversely, the control intervention (povidone–iodine for external lesions) is known to have cytotoxic effects on healing tissues, which may have disadvantaged the control group and contributed to the observed differences. Second, despite multivariable adjustment for age, APACHE II score, and lesion location, residual confounding from unmeasured factors—such as initial wound size and depth, comorbidities (e.g., diabetes mellitus and immunosuppression), nutritional status, and concomitant therapies—cannot be excluded. The numerically higher APACHE II score in the control group (38.38 vs. 33.26), albeit not statistically significant, suggests that the Temporin group may have been somewhat less severely ill at baseline, which could partly explain the superior healing outcomes observed. Third, the heterogeneity of lesion types (oral injuries vs. external skin lesions) limits the ability to draw uniform conclusions across different wound healing environments. Although subgroup analyses suggested consistent benefits, the small sample sizes within each anatomical site precluded robust statistical comparisons. Fourth, the single-centre setting may limit generalisability to other institutions with different patient populations or care protocols. Fifth, the study did not include microbiological assessments to confirm the proposed antibacterial mechanism of Temporin solution (attributed to cetylpyridinium chloride) nor did it evaluate cost-effectiveness, which is essential for informing clinical adoption. Sixth, the uniform 14-day intervention period precludes evaluation of longer-term outcomes, such as wound recurrence or late adverse events. Finally, the absence of observed adverse reactions should be interpreted with caution, given the limited sample size (*n* = 135) and reliance on retrospectively extracted clinical documentation, which may be subject to underreporting. Larger, prospective, randomised, blinded trials with rigorous control of co-interventions, blinded outcome assessment, and inclusion of microbiological and health economic endpoints are needed to establish causality, determine the true effect size, and fully characterise the safety and cost-effectiveness of Temporin atomizable mucosal liquid dressing.

When evaluating the validity of our findings, several methodological considerations must be acknowledged. The internal validity of this study is moderate to low. The retrospective, non-randomised design carries a major risk of confounding by indication. Although the two groups were treated concurrently (minimising temporal bias) and we adjusted for age, APACHE II score, and lesion location, we could not adjust for all potential confounders—such as initial wound size, depth, nutritional status, or comorbidities—due to limitations of retrospective data collection. The lack of blinding introduces performance and detection bias, as patients, caregivers, and outcome assessors were aware of the intervention received. Residual confounding, therefore, remains a significant threat, and causal inferences should be drawn with caution. External validity is limited. As a single-centre study conducted in a Chinese ICU population including only Stage 2 pressure injuries and oral injuries, findings may not be generalisable to other settings, wound types, or populations. Moreover, Temporin is a specific commercial product that may not be widely available internationally, further limiting global applicability. Construct validity is moderate. Healing was defined clinically using standardised NPUAP criteria, reflecting real-world practice. However, the absence of objective quantification methods (e.g., wound photography and planimetry) and microbiological data to support the proposed antibacterial mechanism limits mechanistic interpretation. Lack of blinded outcome assessment further compounds this limitation. In summary, although this study provides preliminary evidence supporting the use of Temporin in critically ill patients, the moderate-to-low internal validity, limited external validity, and moderate construct validity underscore the need for prospective randomised controlled trials with rigorous blinding, comprehensive confounding control, and objective outcome measures to confirm these findings.

In conclusion, this retrospective study found that Temporin atomizable mucosal liquid dressing was associated with improved wound healing outcomes compared with routine care in critically ill patients, with no observed safety concerns. These findings support the need for further prospective investigation. It is recommended that prospective, multicentre, large-sample randomised controlled trials be conducted in the future to further verify the solution’s efficacy and explore its applicability in different populations and different types of injuries.

## Conclusion

This retrospective analysis found that the use of Temporin atomizable mucosal liquid dressing was associated with higher healing rates and shorter healing times compared with routine care in critically ill patients with Stage 2 pressure injuries or oral injuries, with no safety concerns observed. However, due to the retrospective design, lack of blinding, and potential residual confounding, these findings should be interpreted as demonstrating association rather than causation or clinical superiority. The observed differences may be partly attributable to baseline imbalances in disease severity or the cytotoxic effect of povidone–iodine used in the control group. Given the physiological differences between the oral mucosa and epidermis, the results should not be generalised across wound types. Furthermore, the single-centre setting and use of a specific commercial product limit generalisability. Well-designed, multicentre, randomised controlled trials with blinded outcome assessment, objective wound measurement, and longer follow-up are urgently needed to confirm these preliminary findings and establish the true efficacy and cost-effectiveness of this intervention.

## Data Availability

The original contributions presented in the study are included in the article/supplementary material, further inquiries can be directed to the corresponding author.
